# Alloreactive-free CAR-VST therapy: a step forward in long-term tumor control in viral context

**DOI:** 10.3389/fimmu.2024.1527648

**Published:** 2025-01-15

**Authors:** Valentine Wang, Barbara Savoldo, José-Arthur Guimaraes, Gianpietro Dotti, Loïc Reppel, Danièle Bensoussan

**Affiliations:** ^1^ Unité Mixte de Recherche (UMR) 7365 Centre National de la Recherche Scientifique (CNRS), Ingénierie Moléculaire, Cellulaire et Physiopathologie (IMoPA), Université de Lorraine, Nancy, France; ^2^ Lineberger Comprehensive Cancer Center, University of North Carolina, Chapel Hill, NC, United States; ^3^ Centre Hospitalier Régional Universitaire (CHRU) Nancy, Cell Therapy and Tissue Bank Unit, MTInov Bioproduction and Biotherapy Integrator, Nancy, France

**Keywords:** CAR-T, virus specific T cell, CAR-VST, allogeneic, GvHD, graft rejection

## Abstract

CAR-T cell therapy has revolutionized immunotherapy but its allogeneic application, using various strategies, faces significant challenges including graft-versus-host disease and graft rejection. Recent advances using Virus Specific T cells to generate CAR-VST have demonstrated potential for enhanced persistence and antitumor efficacy, positioning CAR-VSTs as a promising alternative to conventional CAR-T cells in an allogeneic setting. This review provides a comprehensive overview of CAR-VST development, emphasizing strategies to mitigate immunogenicity, such as using a specialized TCR, and approaches to improve therapeutic persistence against host immune responses. In this review, we discuss the production methods of CAR-VSTs and explore optimization strategies to enhance their functionality, activation profiles, memory persistence, and exhaustion resistance. Emphasis is placed on their unique dual specificity for both antitumor and antiviral responses, along with an in-depth examination of preclinical and clinical outcomes. We highlight how these advances contribute to the efficacy and durability of CAR-VSTs in therapeutic settings, offering new perspectives for broad clinical applications. By focusing on the key mechanisms that enable CAR-VSTs to address autologous CAR-T cell challenges, this review highlights their potential as a promising strategy for developing effective allogeneic CAR-T therapies.

## 1 Introduction

Autologous Chimeric Antigen Receptor T cell (CAR-T cell) therapy, while highly personalized and effective, faces several significant limitations. The manufacturing process is complex and time-consuming, often taking weeks to harvest, engineer, and expand the patient’s own T cells (1). This delay can be critical for patients with rapidly progressing diseases. Additionally, the quality of autologous T cells can be compromised in heavily pretreated or immunocompromised patients, potentially reducing the efficacy of the therapy (2). The cost associated with the individualized production of autologous CAR-T cells is also substantial, making it less accessible to a broader patient population (between 300 000-400 000$).

In contrast, allogeneic CAR-T cells represent a promising solution to overcome these challenges. Allogeneic CAR-T are derived from “treatment naïve” healthy donors, allowing for the generation of “off-the-shelf” products that can be prepared in advance and made readily available, with a significant reduction of the time from diagnosis to treatment (3). By using a restricted number of donors, production costs are lowered through large-scale manufacturing, making the treatment more accessible. Despite these ideal characteristics, the potential for graft-versus-host disease (GvHD) and the risk of rejection, which limits the efficiency and persistence of allogeneic CAR-T cells, remain significant hurdles. Lymphodepletion and various sophisticated gene modifications have been explored to prevent such complications. However, the alloreactivity of allogeneic CAR-T cells can lead to life-threatening complications, limiting their widespread use (4).

Using virus specific T cells (VST) as a raw material to generate CAR-T cells is an effective way to mitigate some of these drawbacks. Indeed, VST are associated with a low risk of GvHD (5, 6). Moreover, their anti-viral TCR contributes to their prolonged persistence through repeated virus reactivations or restimulations, enhancing the durability and efficacy of the therapy.

After a brief state of the art about allogeneic CAR-T cells, we will describe in the current review, the potential of VST then achievements of CAR-VST therapy, focusing on its development, preclinical research, and clinical applications.

## 2 Allogeneic CAR-T cells

Understanding alloreactivity mechanisms like graft-versus-host disease (GvHD) and graft rejection (GR) is crucial to develop strategies to develop allogeneic CAR-T cells.

### 2.1 Strategies for allogeneic CAR-T cells

To mitigate rejection of infused allogeneic VSTs by recipient-derived immune responses, lymphodepleting chemotherapy or radiotherapy is typically employed to reduce the host’s immune response. Enhancing lymphodepletion prior to CAR-T cell infusion further reduces recipient T cell numbers, creating a more favorable environment for graft acceptance. Another approach involves creating Human Leukocyte Antigen (HLA)-matched cell banks to reduce immunogenicity (7), while gene-editing techniques, such as Clustered Regularly Interspaced Short Palindromic repeats (CRISPR) or Transcription Activator-Like Effector Nuclease (TALEN), are used to knock out HLA class I molecules, thus decreasing T-cell-mediated rejection (8–10). However, since Natural Killer (NK) cells can target cells lacking HLA class I through “missing-self” recognition, overexpressing non-classical HLA molecules, like HLA-E or HLA-G, can protect CAR-T cells from NK cell-mediated lysis (11–14).

To reduce GvHD, researchers have focused on preventing alloreactivity by modifying T cells to minimize their interaction with the recipient’s immune system. Gene editing to knock out the TCR, particularly the TRAC gene, prevents T cells from recognizing and attacking recipient tissues, thus reducing GvHD risk. Technologies like CRISPR/Cas9, Zinc Finger Nucleases (ZFN), and TALEN are instrumental in achieving precise TCR knock-out (4, 15–19). Another approach consists in using non-T cell types- such as Natural Killer cells (20, 21), γδ T cells (22–24), Mucosal-Associated Invariant T (MAIT) cells (25–27), Double Negative T cells (DNTs) (28–31), Cytokine-Induced Killer cells (CIK) (32, 33), invariant NKT (iNKT) cells (34–37), inducible Pluripotent Stem Cell (iPSC) (38–40) and Virus Specific T cells (VST) cells-, as they have less alloreactivity leading to a reduced risk of inducing GvHD. For instance, NK cells provide a potent cytotoxic response regardless TCR involvement, while VST cells leverage prior viral specificity to reduce alloreactivity and minimize GvHD.

With these strategies in place to prevent GvHD risk, the focus now shifts to evaluating the clinical outcomes of allogeneic CAR-T cell therapies and their potential benefits across patient populations.

### 2.2 Clinical outcomes

Recent reviews highlight various strategies for producing allogeneic CAR-T cells using previous cited strategies to disrupt TCR and CD52 genes, minimizing GvHD and rejection risks (41–44). Many *off-the-shelf* products, such as UCART19/ALLO-501, have shown encouraging outcomes, achieving a 48% overall response rate (ORR) in B-ALL and lymphoma with manageable GvHD (45). Advanced trials, like ALLO-501A, report a 67% ORR without GvHD (ALPHA2 (NCT04416984), EXPAND (NCT05714345)) (46). Other candidates targeting CD123, CD22, and BCMA have achieved ORRs around 70% without GvHD (47–49). PBCAR0191 and CTX110 showed high efficacy (up to 83%) in lymphoma and B-ALL even after prior CAR-T failure (50). Innovative approaches, including shRNA-based CYAD-101 and iPSC-derived FT819, have shown good tolerability and stable outcomes (51–53). To address rejection without excessive immunosuppression, gene-editing strategies aim to reduce CAR-T cell immunogenicity. For instance, knocking out β2-microglobulin (β2M) prevents expression of HLA class I molecules, limiting recognition by host T cells. Some products, like PBCAR19B, also express HLA-E, which binds inhibitory receptors on NK cells, reducing NK-mediated lysis (54). Other approaches, such as deleting both β2M and CD70 (as in CTX-130), aim to reduce recognition by both T and NK cells, improving CAR-T persistence in the host.

Building on the advances and challenges of allogeneic CAR-T cell development, we will focus on the strategy of using VST cells as a primary source for CAR-T cells, leveraging their unique immunological properties to improve the safety, persistence, and efficacy of allogeneic CAR-T therapies.

## 3 Virus specific T cells: state of the art

Viral infections, reactivations or diseases remain major complications in immunocompromised patients, including those with primary immunodeficiency or secondary immunodeficiency due to (i) allogeneic hematopoietic stem cell transplantation (allo-SCT), (ii) solid organ transplantation (SOT), (iii) immunosuppressive treatment, or (iv) human immunodeficiency virus infection. Although improvements in the management of viral infections have been made thanks to the implementation of new antiviral drugs, prophylactic and pre-emptive administration and viral load monitoring, in the absence of specific antiviral immunity, antiviral strategies are often ineffective, leading to treatment failure. To address this major limitation, adoptive transfer of virus specific T cells (VST) has been explored.

**Figure 1:**
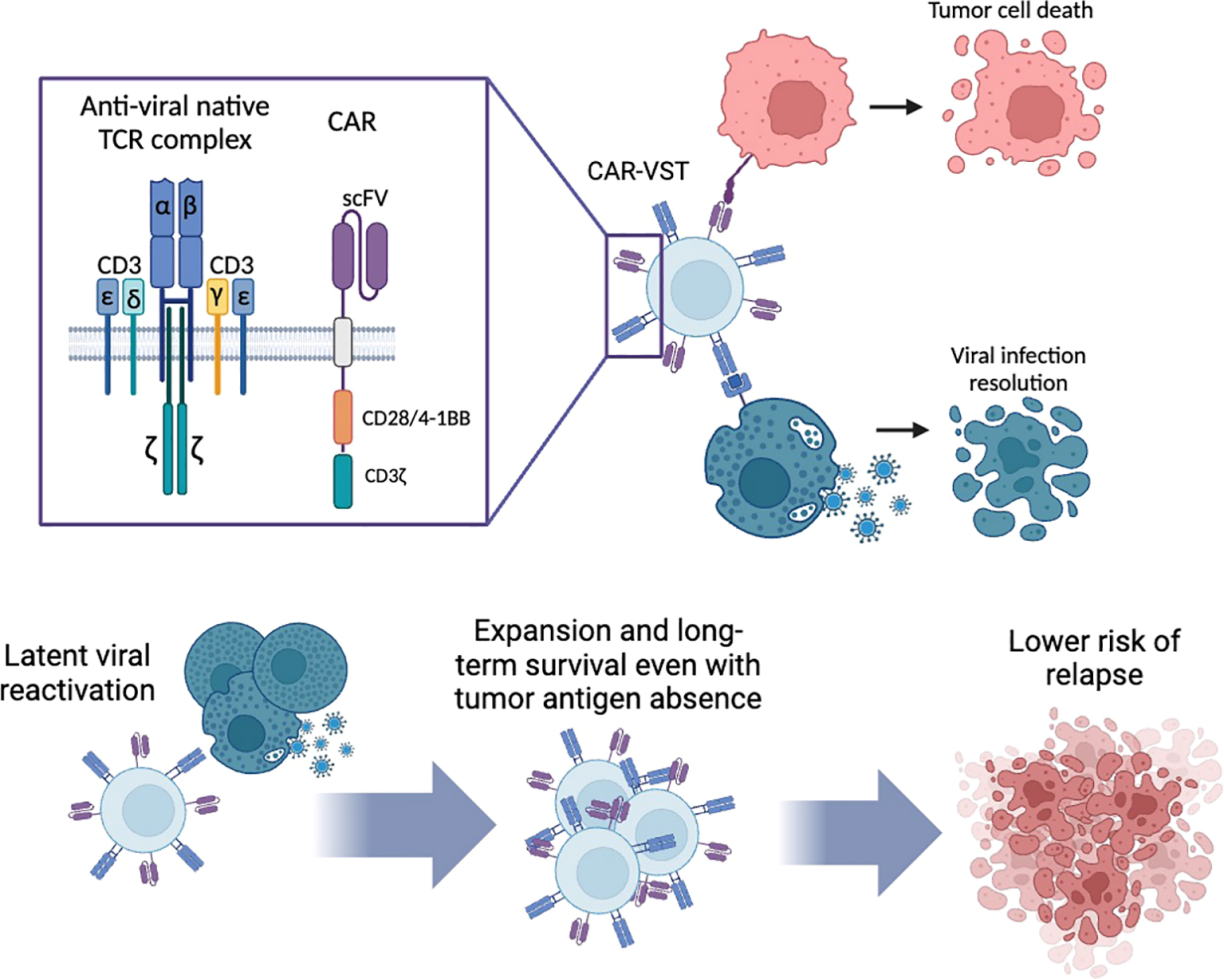
Dual specificity of CAR-VST: antitumoral lysis by the CAR and antiviral lysis via their native TCR. Long-term survival of CAR-VST is expected through the restimulation of the TCR by latent virus reactivation. CAR, Chimeric Antigen Receptor; CD, Cluster of differentiation; VST, Virus Specific T cell. Created with Biorender.com.

VST are isolated from a donor’s lymphocyte pool and require prior immunization of the donor to the target viruses. For example, about 90% of the adult population has prior immunity to Epstein-Barr virus (EBV), while nearly 100% of the adult population in Asia and about 50% in Europe have immunity to cytomegalovirus (CMV) (55). After infusion into the patient, VST proliferate upon encounter with the specific viral antigens presented by the recipient’s HLA molecules, and generate an antiviral immune response. The source of these VSTs can be the allo-SCT donor or a different donor, known as a third-party donor, which can overcome issues associated with the lack of availability of an allogeneic HPC donor for the generation of donor-derived VSTs. In the context of allo-HCT, the use of third-party VSTs allows for immediate access to an antiviral therapeutic product, which can overcome issues associated with limited access to the allo-SCT donor (e.g., lack of donor availability or prolonged manufacturing times in the event of a seronegative donor) (56). Additionally, it can expedite the process in SOT or in case of immunodeficiency, by using a readily available donor or ready-to-use HLA-typed antiviral VST from a bank (**Figure 1**). The qualitative characteristics of generated VST vary depending on the type of donor, the production method and the targeted virus. Currently, two major production strategies are commonly implemented: *ex vivo* expansion of specific VST by cell culture or direct immunomagnetic isolation of VST.

*Ex vivo* expansion relies on the co-culture of peripheral blood mononuclear cells (PBMC) with autologous antigen-presenting cells (APC), such as EBV-transformed lymphoblastoid cell lines (LCL) (57), antigen-pulsed dendritic cells (DC) or, more recently, peptide-loaded APC (58). This method, which requires a minimum of 10 days, allows for the expansion of large numbers of polyclonal VST, containing both CD8 and CD4 T cells.

Immunomagnetic isolation of VST requires the use of a device like the CliniMACS or its automated counterpart, the Prodigy (Miltenyi Biotec, Bergisch-Gladbach, Germany). Enrichment of VST based on IFN-γ secretion involves stimulating PBMC with one or more synthetic peptide pools, and subsequently isolation using the IFN-γ Catchmatrix reagent (Cytokine Capture System, Miltenyi Biotec). This procedure can be completed in 2 days including leukapheresis. Although a very small number of cells is often obtained through this process, VST are polyclonal and contain both CD8 and CD4 T cells (59). Another way to target VST before immunomagnetic sorting consists in using HLA restricted-multimers, which offers a highly specific approach by binding the TCR on specific T cells. However, isolated VST are usually composed either of a CD8 or a CD4 T-cell clone, depending on the MHC molecule used in the multimer (MHC class II multimers are still rarely available), leading to a very low number of VST, often lacking CD4 T cell support over time (60).

The use of *ex-vivo* expanded specific CD8+ T-cells from the initial donor was first proposed Riddell et al. in 1992 as a VST strategy to treat CMV reactivation after allo-SCT (61). Subsequently EBVST generated from donor derived PBMC stimulated with irradiated autologous LCL were utilized to prevent EBV reactivation (62, 63). In 2006, freshly immunomagnetically-isolated AdV-VST from allo-SCT donor leukapheresis without any prior expansion, successfully control in 4 out of 5 evaluable patients with AdV infection-related complications (59). The need for a fast, efficient and safe treatment for early post-transplant viral infections prompted the generation of VST from third-party donors. This development broadened the applicability of VST due to their low capacity to induce alloreactivity even when using HLA-mismatched donors (5, 6, 64). Currently, off-the-shelf, potentially multi-target VSTs represent a promising therapy for both early and late-stage viral infections in immunocompromised patients, provided that a compatible VST cell line is available (58, 65, 66).

To date, data from more than 50 clinical studies (phase I, I/II and II) currently available provide encouraging results, both regarding antiviral efficiency and tolerance (67). Safety studies reported that a minority of patients experienced no to low side effects related to VST therapy -specifically GvHD, Cytokine Release Syndrome (CRS), infusion toxicity, transplant-associated thrombotic microangiopathy, graft failure, and genitourinary complications- none of which were serious (grade I/II), allowing for a short monitoring period of one hour after VST infusion (68, 69).

The reported risk of post-administration GvHD is relatively low, around 10%, regardless of the antiviral VST type and donors, including third parties with partial HLA compatibility (70). Among the reported cases of GvHD, it appears that most of them are reactivations (2/3). However, it remains impossible to discriminate between the effects of the VST themselves and modulation of immunosuppressive drugs in patients waiting VST infusion (5). Nevertheless, heterologous immunity, which refers to the cross-reactivity of VST with allo-antigens in an allogeneic context, remains rarely observed, despite being a theoretical concern. This was first reported in the context of allo-SCT, with low GvHD incidence, whereas VST presented cross-reactivity with recipient HLA molecules *in vitro* (71). More recently, a lack of association between the presence of cross-reactive VST and decreased graft survival has been systematically observed in SOT patients (72). Several explanations have been proposed, including a lower avidity of VST TCR for the allogeneic epitope compared to the viral epitope, and the role of immunosuppressive regimens in transplanted patients.

Regarding antiviral efficacy, 65-90% of patients achieved a partial or complete antiviral response across various clinical studies (73). Different reasons have been suggested to explain this range. First, the delay between viral infection and VST infusion. In line with this assessment, our team observed a strong impact of a high viral load (>5 log) on overall survival, regardless of the involved virus, suggesting that VST should be considered as soon as a patient experience a chemo-refractory viral infection following allo-SCT (6). Moreover, the matching between VST and the patient appears to be more critical for the viral restricting alleles than for the overall degree of match *per se* (74). Last but not least, a specific antiviral immune reconstitution was frequently associated to the decrease or clearance of the viral load (59). This means that all the conditions must be met for *in vivo* VST expansion, particularly a moderate immunosuppression, given the role played by corticosteroids as previously reported in an *in vitro* study (75).

However, up to now, no phase III clinical efficacy study has been published. A randomized, controlled study in a large cohort of patients comparing antiviral treatment alone to antiviral treatment combined with VST will be helpful to confirm safety and efficacy. To this end, the results from Trace (TRansfer of Adenovirus, Cytomegalovirus and Epstein-Barr virus specific-T cells -NCT04832607), a European comparative study, are highly anticipated.

The persistence for up to 9 years of functional VST has been reported (57). Current data suggest that, rather than the total amount of VST infused, the frequency of different lymphocyte subpopulations (especially memory T stem cells (Tscm)) (5) is crucial for the *in vivo* expansion of VST and the persistence of the antiviral response (76),. Indeed, Gattinoni and colleagues identified distinct T cell subsets with differing potential for persistence and therapeutic efficacy in adoptive immunotherapy (77). These subsets include naive T cells (Tn), central memory T cells (Tcm), effector memory T cells (Tem), and stem cell memory T cells (Tscm). Tscm are of significant interest due to their superior longevity, self-renewal capacity, and ability to differentiate into other T cell subsets, making them ideal for adoptive cell therapies. Our team reported that immunomagnetic sorted VST contained Tscm, although poorly represented (around 1%), which could be sufficient to allow for (i) differentiation into Tcm, Tem and Teff subsets according to the linear developmental model, and (ii) maintenance of the proportion of IFN-γ+ cells among Tscm (78).

## 4 Development of CAR-VST as an alternative for allogeneic CAR-T cell products

### 4.1 Virus specific T cells advantages

The generation of VST opens avenues for the development of CAR-VST, offering distinct advantages in terms of quantity and subpopulation diversity. The development of CAR-VST presents a promising alternative to conventional allogeneic CAR-T cell therapy, offering a versatile and potentially more accessible therapeutic option. Moreover, CAR-VSTs may provide additional regulatory and safety benefits compared to TCR knockdown strategies using CRISPR-Cas9 or other gene modification techniques, as these approaches carry a potentially increased risk of genotoxicity and malignant transformation (79, 80). The different methods to generate VST influence the characteristics of the resulting CAR-VST.

CAR-VST maintain robust antitumor efficacy due to their dual specificity. They are capable of targeting both tumor cells through their CAR and viral infected cells *via* their native TCR. This dual targeting is particularly beneficial for sustained and targeted therapeutic responses.

One of the major advantages VST can provide is the long-term persistence by the restimulation of their native TCR. This can occur through the spontaneous reactivation of latent viruses, making VST against latent viruses such as EBV, CMV and AdV ideal candidates. Alternatively, CAR-VST can be restimulated on demand using existing or manufactured vaccines against viruses like VZV or CMV, ensuring continuous expansion and activity. Unlike traditional CAR-T cells, which often suffer from limited efficiency and persistence, CAR-VST are expected to benefit from the continued expression of a functioning TCR.

Moreover CAR-VST are associated with a low incidence of GvHD, a common complication expected with traditional allogeneic CAR-T cells. As mentioned previously, the low or absence of alloreactivity is due to inherent properties of VST, which have been amply demonstrated in clinical trials (71, 72).

However, the risk of rejection remains a challenge. Different strategies can be employed to address this drawback. One approach involves the engineering of these cells to limit their expression of HLA molecules, thereby reducing their immunogenicity. However, this strategy makes CAR-T cells susceptible to NK killing. An alternative strategy is based on selecting an intrafamilial third-party donor to provide high-quality cells with reduced rejection risks. While this option is not suitable for *off-the-shelf* production and does not lower costs, it offers a reliable source of at least semi-compatible cells.

### 4.2 VST investigated to produce CAR-VST

**Figure 2:**
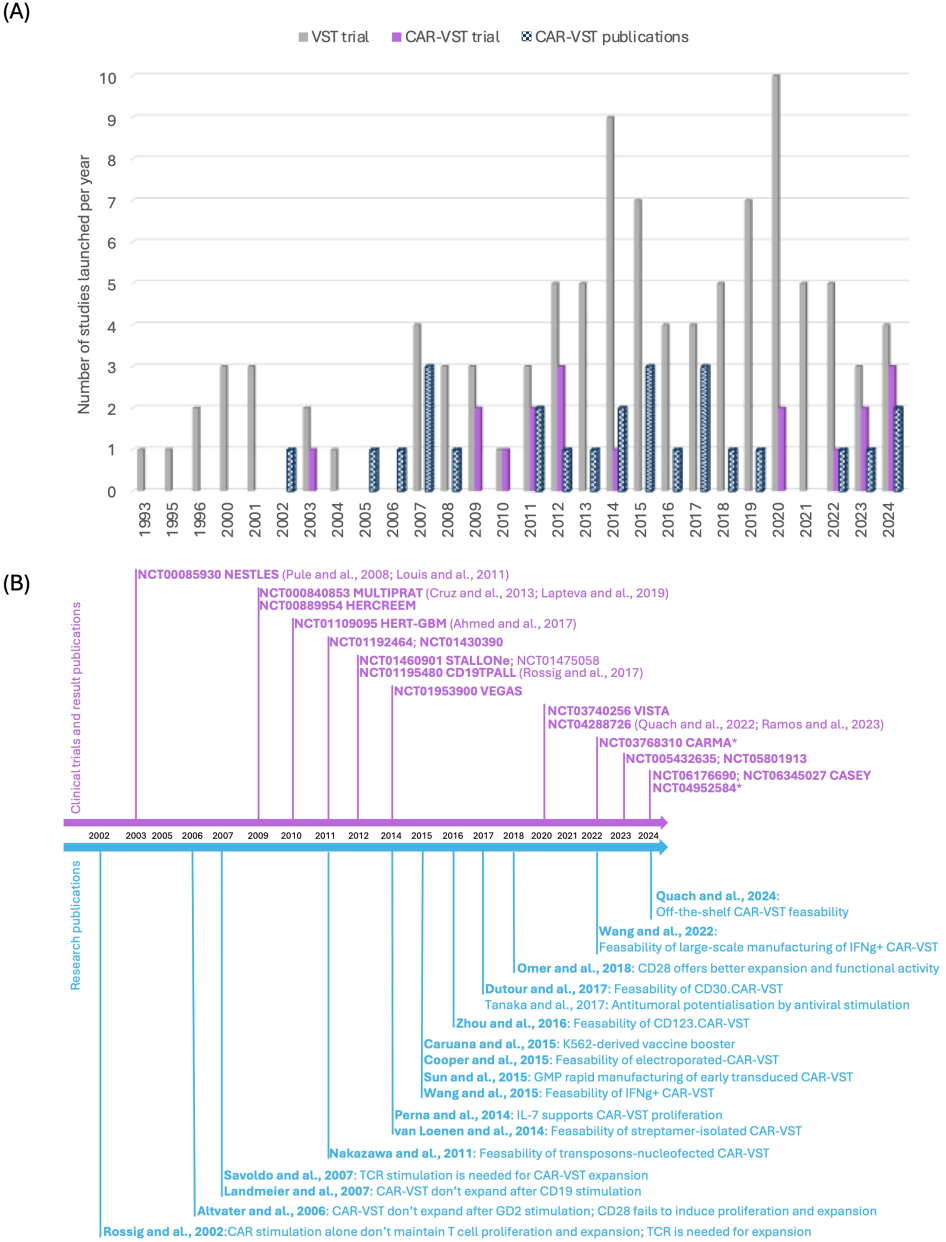
State of the art of CAR-VST in pre-clinical and clinical studies. **(A)** Publications on pre-clinical and clinical trials on CAR-VST and start year of clinical trials by year (clinicaltrial.gov, May 2024); **(B)** Relevant articles on CAR-VST pre-clinical results (blue) and clinical results (purple) by year. CAR, Chimeric Antigen Receptor; CD, Cluster of differentiation; GMP, good manufacturing practice; IFNγ, Interferon gamma; TCR, T cell receptor; VST, Virus Specific T cell.

Clinical trials involving VST began to emerge significantly in the early 2000s (**Figure 2**). Initially, research on VST primarily focused on treating viral infections and their role in the context of transplantation. The introduction of CAR-VST into clinical research was initially relatively slow, with only a few pioneering studies before 2010. However, beginning in the 2010s, there has been a notable increase in the number of clinical trials. Simultaneously, there has been a progressive increase in research publications on CAR-VST, reflecting a growing interest in this promising therapy.

Several leading institutions are advancing the research and development of CAR-VST therapies, primarily in the USA.

The Center for Cell and Gene Therapy at Baylor College of Medicine in Houston, USA, has been extensively investigating CAR-VSTs targeting antigens such as GD2, CD19, CD30, and HER2. Their work involves the use of various cytokines and transduction methods to enhance the expansion and persistence of these cells. Collaborating with other institutions, they focused on improving both *in vitro* and *in vivo* antitumor activity. At the City of Hope in Duarte, California, researchers have used CD19-targeting CAR-VSTs, employing innovative vaccination strategies to boost efficacy and persistence.

In Europe, the University Children’s Hospital Münster in Germany is working on GD2.CAR-VST, addressing challenges in CAR-VST expansion and co-stimulation requirements. INSERM U590 at Centre Léon Bérard in Lyon, France, is developing CD33.CAR-VST, maintaining a memory effector phenotype with demonstrated functional antitumor and antiviral activities. These institutions collectively contribute to the evolving field of CAR-VST therapy, aiming to enhance the safety, specificity, and therapeutic efficacy of cancer immunotherapies. These studies are summarized in **Tables 1**, **2**.

**Table 1:** Pre-clinical studies on CAR-VST.

Team	Cells	VST sorting	Transduction	References
Center for Cell and Gene Therapy, Baylor College of Medicine, Houston, USA.	GD2.CAR-EBVST	Three stimulations by irradiated autologous EBV-LCL	Anti-OKT3 anti-CD28 antibodies After the third stimulation of irradiated LCL Retrovirus, retronectin	(81)
	CD30.CAR-VST CD28	Three stimulations by irradiated autologous EBV-LCL+IL-2 ADV or CMV	After the third stimulation of irradiated LCL Retrovirus, retronectin	(82)
	iCas9.GD2.CAR-CMVST CD28	Stimulation with autologous DC loaded with pp65 pepmix	After the second stimulation of irradiated LCL Retrovirus, retronectin	(83)
	GD2.CAR-EBVST	Three stimulations by irradiated autologous EBV-LCL	Early and late transduction Retrovirus, retronectin	(84)
	HER2-EBVST HER2-EBVST.iCD19 CD28	Stimulation with irradiated autologous EBV-LCL	Nucleofection with transposons	(85)
	GD2.CAR-VST CD19.CAR-VST Either CD28 or 41BB	Stimulation with autologous DC, PBMC and peptide-loaded-K562 (VZV or EBV)	Retrovirus, retronectin	(86)
	CD30.CAR-EBVST 2^nd^ generation CD28	PBMCs were depleted of CD45RA positive cells by magnetic column separation, then stimulated with EBV pepmixes	Retrovirus, retronectin	(87)
Center for Cell and Gene Therapy, Baylor College of Medicine, Houston, USA + collaborators	GD2.CAR-EBVST GD2.CAR-EBVST.IL7R CD28	Stimulation with irradiated autologous EBV-LCL	Retrovirus, retronectin	(88)
	GD2.CAR-VZVST 3^rd^ generation	PBMCs pulsed with overlapping peptide libraries spanning selected VZV antigens	Retrovirus, retronectin	(89)
	CD123.CAR-VST (AdV, CMV or EBV) CD28	Stimulation with autologous peptide-pulsed-DC+CD3/CD28 antibodies 1 μg/ml	Retrovirus, retronectin	(90)
Departments of Hematology and Hemamiddleoietic Cell Transplantation, City of Hope, Duarte, California	CD19.CAR.CD8- MP1.VST (Influenza) 1st generation	Stimulations by irradiated autologous LCL	Electroporation	(91)
	CD19.CAR-CMVST CD28	IFN-γ immunomagnetic selection after pp65 stimulation	Lentivirus (MOI=3), protamine sulfate	(92)
	CD19.CAR-CMVST CD28			(93)
University Children’s Hospital Münster, Department of Paediatric Haematology and Oncology, Münster, Germany.	GD2.CAR-EBVST With or without CD28	Stimulation with irradiated autologous EBV-LCL	Retrovirus, retronectin	(94)
	GD2.CAR-VZVST CD19.CAR-VZVST With or without CD28	Stimulation with VZV lysates+irradiated autologous PBMC	Retrovirus, retronectin	(95)
INSERM U590/Equipe Cytokines et Cancer, Centre Léon Bérard, 69373 Lyon Cedex 08, France.	CD33.CAR-EBVST CD28	Stimulation with irradiated autologous EBV-LCL	Retrovirus	(96)

CAR, Chimeric Antigen Receptor; CD, Cluster of differentiation; CMV, Cytomegalovirus; CMVST, Cytomegalovirus Virus Specific T cell; DC, Dendritic Cell; EBV, Epstein-barr virus; EBVST, Epstein-barr Virus Specific T cell; GD2, disialoganglioside; HER2, Human Epidermal Growth Factor Receptor 2; IFNγ, Interferon gamma; IL, Interleukin; LCL, Lymphoblastoid Cell Line; MP-1, influenza A Matrix Protein 1; PBMC, Peripheral Blood Mononuclear Cell; TCR, T cell receptor; VST, Virus Specific T cell; VZV, Varicella Zoster Virus.

**Table 2:** Clinical trials and published results about CAR-VST (clinicaltrial.gov).

Team	Target and conditions	Virus specificity	Cell source	Additional treatment	Status	Phase	References; acronym	Start year
Baylor College of Medicine, Houston, USA	CD19 B-cell malignancies	EBV CMV, EBV, Adv, BKV and HHV-6	Allogeneic Allogeneic	Following allogeneic HSCT	Ongoing Withdrawn	1	NCT00840853; MULTIPRAT (68) NCT03768310; CARMA* NCT unknown (97);	2009 2022*
	CD30 Lymphoma	EBV	Allogeneic Autologous Allogeneic Allogeneic	bank of 7 lines from healthy donors IL7 receptor overexpressed	Recruiting Ongoing Withdrawn Not yet recruiting	1	NCT04288726 (98) NCT01192464 NCT04952584* NCT06176690	2020 2011 2024* 2024
	HER2 solid tumors	EBV ADV	Autologous Autologous	TGF-B resistance Oncolytic viruses	Completed Recruiting	1	NCT00889954; HERCREEM (no results so far) NCT03740256; VISTA	2009 2020
	CD70 B-cell malignancies	EBV	Autologous		Not yet recruiting	1	NCT06345027; CASEY	2024
Baylor College of Medicine, Houston, USA & collaborators	GD2 sarcoma Neuroblastoma	VZV EBV	Autologous Autologous	Vaccine	Ongoing Ongoing	1	NCT01953900; VEGAS NCT00085930; NESTLES (99, 100)	2014 2003
	HER2 Glioblastoma	CMV	Autologous		Completed	1	NCT01109095; HERT-GBM (101)	2010
Children’s Mercy Hospital Kansas City and Baylor College of Medicine	GD2 Neuroblastoma	CMV ADV EBV	Allogeneic		Completed	1	NCT01460901; STALLONe	2012
Memorial Sloan Kettering Cancer Center	CD19 B-cell malignancies	EBV	Allogeneic		Ongoing	1	NCT01430390	2011
City of Hope Medical Center, California	CD19 B-cell malignancies	CMV (vaccine) CMV (vaccine)	Autologous Autologous	Vaccine Vaccine	Recruiting Recruiting	1	NCT05432635 NCT05801913	2023 2023
NCI et Fred Hutchinson Cancer Research Center	CD19 B-cell malignancies	CMV EBV	Allogeneic	Following allogeneic HCST	Completed	1/2	NCT01475058 (unpublished data)	2012
University College, London	CD19 B-cell malignancies	EBV (vaccine)	Allogeneic		Unknown	1/2	NCT01195480; CD19TPALL (102)	2012

*withdrawn clinical trials; AdV, Adenovirus; BKV, Bk virus or John Cunningham virus; CMV, Cytomegalovirus; EBV, Epstein-Barr virus; HHV, human herpesvirus; HSCT, Hemamiddleoietic stem cell transplantation; IL, Interleukine; TGF, Transforming growth factor-beta; VZV, Varicella Zona Virus.

## 5 Pre-clinical research on CAR-VST

### 5.1 Cell manufacturing

All the characteristics are summarized in **Table 1**.

#### 5.1.1 VST manufacturing

**Figure 3:**
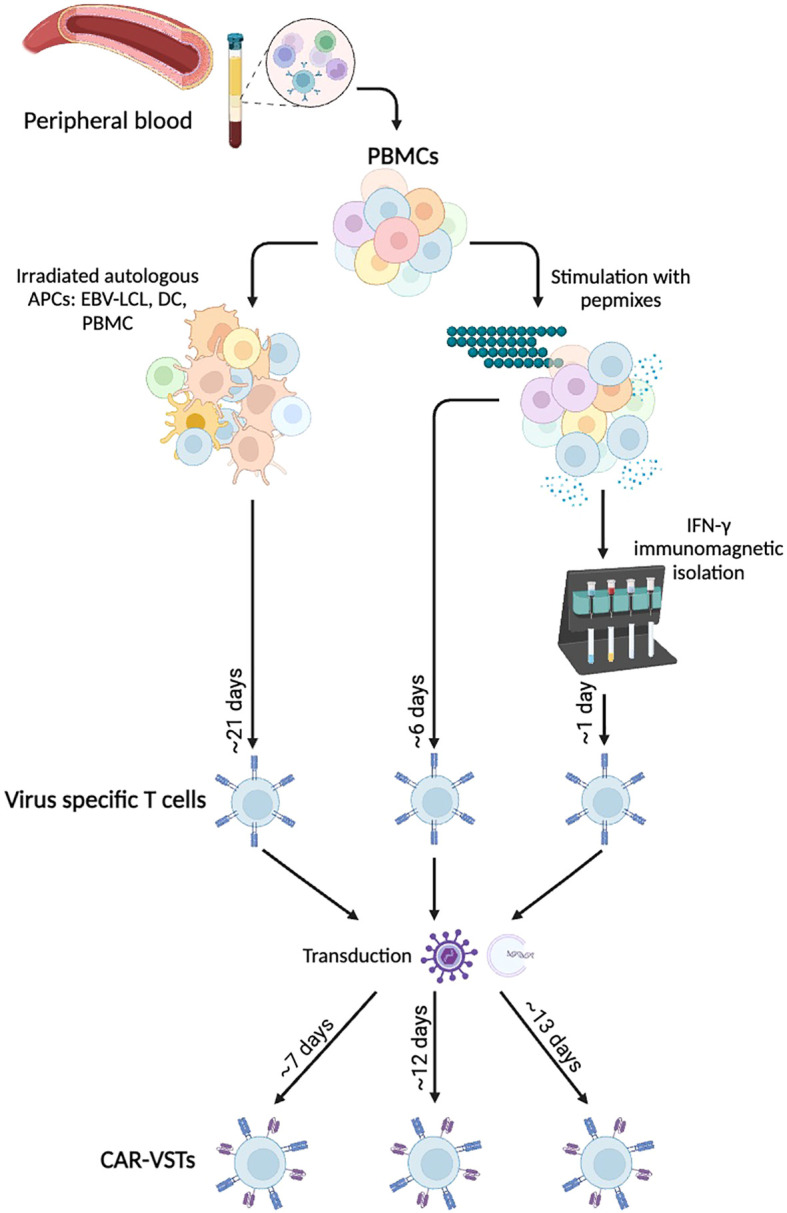
Manufacturing methods for *ex vivo* CAR-VST: VSTs are mainly produce either by coculture with Antigen Presenting Cells (APCs) or after peptide pool stimulation with or without immunomagnetic IFN-γ selection. Viral transduction or electroporation are performed to express the CAR transgene, leading to bi-specific CAR-VSTs. APC, Antigen Presenting Cell; CAR, Chimeric Antigen Receptor; DC, Dendritic Cell; EBV, Epstein-barr virus; IFNγ, Interferon gamma; LCL, Lymphoblastoid Cell Line; PBMC, Peripheral Blood Mononuclear Cell; VST, Virus Specific T cell. Created with BioRender.com.

The manufacturing of CAR-VST involves several critical steps to ensure the effective generation and expansion of these therapeutic cells (**Figure 3**). As mentioned in section 1, two approaches are consistently used to generate VSTs, which we will briefly summarize here.

##### 5.1.1.1 Coculture methods

Most of the CAR-VST reported in the literature are generated from VST obtained in co-culture of PBMC with autologous APC. This approach has proven effective for manufacturing large quantities of VST, which is advantageous for producing multiple batches. However, this method requires long expansion periods, often taking at least three weeks with repeated restimulations, which can also lead to more differentiated and exhausted T cells. As autologous antigen presenting cells LCL (81, 94, 96), dendritic cells (83, 90) or PBMC loaded with viral antigens like VZV have been used (95). Alternatively, Quach et al. directly stimulated CD45RA-depleted PBMC with pepmixes specific to EBV antigens (87), resulting in a robust expansion of VST that showed response to EBV stimulation. Recombinant human interleukine-2 (IL-2) is the most common cytokine promoting T cell survival and proliferation. However, IL-2 is also known to induce a terminal effector phenotype which is correlated with strong cytotoxicity but short-term lifespan (77). Other cytokines like IL-4, IL-7 and/or IL-15 are currently under investigations to promote VST expansion and a more naïve phenotype (84, 87).

##### 5.1.1.2 Immunomagnetic isolation

Immunomagnetic selection is used as an alternative method. In their studies, Wang and colleagues performed nine selection processes using PBMC from eight healthy CMV-seropositive donors. They successfully enriched IFN-γ+ T cells from pre-enrichment levels of 0.8 ± 0.5% to post-selection levels of 76.3% ± 11.6% (92, 93). The freshly isolated IFN-γ+, CMVST consisted in polyclonal CD8+ (44.0% ± 21.0%) and CD4+ T cells (49.8% ± 21.2%). The small number of sorted cells required an additional expansion phase, and further research is urged to enrich for naive and memory cells, rather than the effector phenotype so far obtained.

Overall, each procedure has distinct advantages and limitations. Coculture with LCL, DC or APC is time-consuming and often labor-intensive but produces high cell numbers with robust expansion. Immunomagnetic sorting is a rapid method enriching for highly specific VSTs; however, it leads to a low number of VST, requiring an additional expansion phase. Each method impacts the final VST product’s characteristics, balancing the trade-offs between efficiency, specificity, and scalability to optimize therapeutic efficacy against viral infections and malignancies.

#### 5.1.2 CAR-VST manufacturing

The diversity in CAR sequences, costimulatory molecules and the inclusion of transgenes for cytokine production contributes to the significant variability in CAR expression levels, the extent of CAR-VST activation and their overall functionality. We will report hereafter the targets and the vectors that have been studied up to now in CAR-VST and will discuss later the different improvements in the construct.

Regarding the targets, both well-established and innovative targets are investigated in CAR-VST studies. The CD19 target was the most widely studied to treat B-cell malignancies (3, 91, 92). Several other targets have been investigated including: (i) the disialoganglioside GD2 in solid tumors, especially in glioblastoma and neuroblastoma (81, 83, 84, 86, 88, 89, 94, 95), (ii) HER-2 an antigen expressed in a range of tumors such as breast cancer, lung cancer and ovarian cancer (85) (iii) CD30, a molecule highly and consistently expressed on malignant Hodgkin Reed-Sternberg cells (82, 87, 96), (iv) the CD33 molecule expressed on acute myeloid leukemia blasts (96), as well as (v) the CD123 molecule (90).

The transduction of VST is often the most critical step of the manufacturing process (**Table 3**). Retroviral vectors have been widely used because of their ability to integrate transgenes effectively into the host genome. Retronectin-coated-plates are usually employed to enhance virus and cells contacts, with spinoculation utilized to maintain virus adherence and contact. A large range of transduction efficiency is described in the literature, extending from 10.2% in the first studies to 75%. Increased transgene expressions are observed over time in culture, following restimulations (87), or when transduction is performed early (3 days) after the first stimulation of VST generated with coculture method (84). Lentiviral vectors have also been used and they offer the advantage of transducing both dividing and non-dividing cells, enhancing the flexibility and efficiency of CAR-VST manufacturing. Only one team has described results of CAR-VST produced thought a lentiviral transduction, with increased CAR expression from 8% to 46% after 2 rounds of stimulation (N=3) in a first study and 27.0 ± 14.2% CAR (N=9) in their second study (92, 93).

**Table 3:** CAR-VST manufacturing: transduction strategies.

Team	Cells	Transduction	Transduction efficiency and main results	References
Center for Cell and Gene Therapy, Baylor College of Medicine, Houston, USA.	GD2.CAR-EBVST	Anti-OKT3 anti-CD28 antibodies After the third stimulation of irradiated LCL Retrovirus, retronectin	16.5% CAR expression (N=4)	(81)
	CD30.CAR-VST CD28	After the third stimulation of irradiated LCL Retrovirus, retronectin	26 ± 11% CAR expression (N=8)	(82)
	iCas9.GD2.CAR-CMVST CD28	After the second stimulation of irradiated LCL Retrovirus, retronectin	35-65% CAR expression (N=9)	(83)
	GD2.CAR-EBVST	Early and late transduction Retrovirus, retronectin	Early-and late transduced VSTs was 55 ± 4% and 22 ± 5% respectively (N=6)	(84)
	HER2-EBVST HER2-EBVST.iCD19 CD28	Nucleofection with transposons	47.9 ± 15.5% for HER2.CAR-VST (N=3) 36.4 ± 12.6% for HER2.CAR-VST.iCD19 Long term and stable expression *in vitro* (120 days)	(85)
	GD2.CAR-VST CD19.CAR-VST Either CD28 or 41BB	Retrovirus, retronectin	52-75% CAR expression (N=7)	(86)
	CD30.CAR-EBVST 2^nd^ generation CD28	Retrovirus, retronectin	CD30.CAR expression increased from 40.59% ± 15.76% on day 8, up to 87.25% ± 6.9% at the end of culture (N=3)	(87)
Center for Cell and Gene Therapy, Baylor College of Medicine, Houston, USA + collaborators	GD2.CAR-EBVST GD2.CAR-EBVST.IL7R CD28	Retrovirus, retronectin	64 ± 3% for GD2.CAR (N=5) 34 ± 9% for GD2.CAR.IL7	(88)
	GD2.CAR-VZVST 3^rd^ generation	Retrovirus, retronectin	53.1% ± 7.7% of VZVSTs from naturally infected donors and 44.6% ± 14.8% of VZVSTs from immunized donors (N=3)	(89)
	CD123.CAR-VST (AdV, CMV or EBV) CD28	Retrovirus, retronectin	>30% CAR expression (data not shown)	(90)
Departments of Hematology and Hemamiddleoietic Cell Transplantation, City of Hope, Duarte, California	CD19.CAR.CD8- MP1.VST (Influenza) 1st generation	Electroporation	96% CAR expression (N not specified)	(91)
	CD19.CAR-CMVST CD28	Lentivirus (MOI=3), protamine sulfate	From 8% CAR expression post transduction to 46% after 2 rounds of stimulation (N=3)	(92)
	CD19.CAR-CMVST CD28		27.0 ± 14.2% CAR expression (N=9)	(93)
University Children’s Hospital Münster, Department of Paediatric Haematology and Oncology, Münster, Germany.	GD2.CAR-EBVST With or without CD28	Retrovirus, retronectin	21-28% for GD2 (N=3) 26-40% For GD2.CD28	(94)
	GD2.CAR-VZVST CD19.CAR-VZVST With or without CD28	Retrovirus, retronectin	46 ± 14% CAR expression (N=4)	(95)
INSERM U590/Equipe Cytokines et Cancer, Centre Léon Bérard, 69373 Lyon Cedex 08, France.	CD33.CAR-EBVST CD28	Retrovirus	35 ± 4% CAR expression stable for 1month (N=6)	(96)

CAR, Chimeric Antigen Receptor; CD, Cluster of differentiation; CMV, Cytomegalovirus; CMVST, Cytomegalovirus Virus Specific T cell; DC, Dendritic Cell; EBV, Epstein-barr virus; EBVST, Epstein-barr Virus Specific T cell; GD2, disialoganglioside; HER2, Human Epidermal Growth Factor Receptor 2; IFNγ, Interferon gamma; IL, Interleukin; LCL, Lymphoblastoid Cell Line; OKT3, Orthoclone-muromonab-CD3; PBMC, Peripheral Blood Mononuclear Cell; VST, Virus Specific T cell; VZV, Varicella Zoster Virus.

Transposon systems and electroporation have also been employed as virus-free transduction methods. Nakazawa et al. implemented the Piggy bac-transposon system for transducing EBVST, achieving 47.9% ± 15.5% transduction efficiency for HER2-CAR (N=3) (85). Electroporation, thought electric pulses to introduce DNA into cells, offers a rapid and versatile approach for CAR transduction. Cooper et al. also used electroporation to transduce MP1-specific T cells with a CD19.CAR plasmid, achieving 96% CAR expression (N not specified) (91).

In summary, each transduction method has its unique advantages and challenges. Retroviral and lentiviral vectors are highly efficient but can raise safety consideration related to insertional mutagenesis. Moreover, rare T-cell malignancies were reported from autologous marketed CAR-T cells without evidence of the correlation with integration of the CAR transgene (103, 104) or with derived clonal hematopoiesis (105).While transposon systems are of interest as they provide stable gene integration without the theoretical risks associated with viral vectors, it is important to note the potential risks of malignant transformation associated with both virally transduced and transposon-generated CAR T cells (106).

#### 5.1.3 *In vitro* evidence of bi-specific functionality of CAR-VST

The functional activity of CAR-VST is critical for their therapeutic efficacy. This section summarizes the functional assays and outcomes across the previously mentioned studies, focusing on common results and comparable methodologies.

Preclinical studies show that CAR-VST efficiently lyse tumor cells expressing the targeted tumor antigen, underscoring specific MHC-independent killing. This is true across various CAR, including CD30, GD2 and HER2. Specific lysis rates can vary, but highly enhanced killing compared to non-transduced VST or those targeting irrelevant antigens is consistent. For example, Savoldo et al. and Tanaka et al. reported around 50-58% lysis rates at 20:1 E/T ratio against tumor cells expressing the CAR-targeted antigens and against virus infected cells (see below) (82, 89). Thus, CAR-VST exhibited the dual capacity to lyse both types of targets effectively in cytotoxicity assays. Blocking experiments with monoclonal antibodies against the CAR-targeted antigen confirmed the specificity of the CAR-mediated killing (81, 82, 96). In addition, these CAR-VST did not exhibit cytotoxicity against autologous healthy cells or “non infected” cells (82). Several studies reported that CAR-VST maintained their cytotoxic and cytokine-secreting capabilities over extended culture periods. For instance, Savoldo et al., and Landmeier et al., observed stable and potent antitumor activity in long-term co-cultures (45 days), with CAR-modified T cells effectively eliminating tumor cells and proliferating in response to antigen exposure (82, 95). In addition, it was demonstrated that CAR-VST retained the ability to secrete multiple effector molecules, such as IFNγ, granzyme B and TNF-α, upon activation. Studies by Quach et al., Dutour et al., and Landmeier et al., demonstrated that the poly-functionality of these CAR-VST is preserved post-transduction, indicating that CAR expression does not compromise their broader immune functions (87, 95).

CAR-VST also demonstrated effective lysis of virus-APC, comparable to non-transduced VST targeting the same viruses. For instance, EBVST transduced with CAR retained their ability to lyse EBV-infected cells, showing overall comparable efficiency compared to non-transduced VST. For example, in studies by Rossig et al., and Savoldo et al., CAR-VST lysed autologous LCL effectively, maintaining their MHC-restricted killing capacity (81, 82). This dual functionality of CAR-VST was confirmed by their ability to produce IFN-γ either in ELISPOT assays and intracellular cytokine staining and to proliferate either upon stimulation with specific viral peptides or CAR-targeted tumor cells (90, 93). However, this capability was not consistently observed across all studies. Rossig et al. and Savoldo et al. reported that CAR stimulation alone was inadequate to maintain T cell proliferation and expansion (81, 82). Similarly, Landmeier et al. observed that CD19-CAR-VST did not expand after stimulation with a CD19+ cell line (95).

Overall, CAR-modified VSTs exhibit robust dual functionality, effectively targeting both virus-infected and tumor cells through their TCR and CAR engagement, respectively. These cells maintain their cytotoxicity and cytokine production, making them safe and potent agents for adoptive immunotherapy. However, the proliferation of CAR-VSTs appears to be suboptimal after CAR engagement only, suggesting that their expansion may depend on additional factors, like the presence of adequate costimulatory molecules.

#### 5.1.4 *In vivo* evidence of antitumor efficacy of CAR-VST

*In vivo* evidence of tumor lysis has been assessed in immunocompromised mice models like SCID mice (82, 85) and more recently in NOD SCID mice (96), usually, relying on a FFluc or GFP-FFluc labeled-tumor cell line expressing the antigen of interest. Tumors have been engrafted either intraperitoneally, intra-tumor or intra-venously, with mice receiving CAR-VST or non-transduced VST as a negative control at tumor progression, following the same delivery routes. A study showed that CAR-VST effectively controlled tumor progression for more than two weeks (82) and this protection was further enhanced when they received additional costimulation from autologous EBV-LCL. In contrast, mice receiving control EBVST showed increased tumor growth regardless of costimulation. Similar issues were observed in the other studies. For example mice treated with HER2.CAR-VST had a significantly longer survival, in a brain tumor model (85). In another study, CAR-VST could also be identified by immunohistochemistry at the tumor site, indicating their ability to localize at the tumor and affect the tumor microenvironment (96). Similarly, Savoldo et al., used VST transduced with the GFP-FFluc vector for *in vivo* tracking and showed that both non-transduced (NT-) and CD30.CAR-EBVST localized at the tumor site by day 7 post-infusion and expanded significantly over the next two weeks. This expansion was confirmed to be antigen-dependent, as the bioluminescence signal was significantly lower in mice with EBV+ HLA-mismatched tumors. Although immunodeficient mice have limitations, such as not allowing the study of VST interactions with other immune cells, they offer strong evidence of the potency and dual potential of these cells when humanized.

### 5.2 Strategies to improve CAR-VST functions

#### 5.2.1 Role of endogenous TCR signaling

Signaling through the native TCR/CD3 complex is crucial for the robust activation of CAR-VST. The engagement of the TCR with its specific antigenic peptide presented by MHC molecules on APCs provides a strong and physiologically relevant activation signal. This signaling pathway ensures that T cells, including CAR-VST, maintain their antigen specificity and effector functions. Moreover, some studies have demonstrated that activation of CAR-VST through the CAR alone, although promoting effective antitumor activity, does not fully recapitulate proliferation that occurs through the TCR engagement. In the following section, we will summarize these findings, highlighting the differences in signaling outcomes between CAR and TCR activation.

##### 5.2.1.1 Importance of native TCR signaling for CAR-VST proliferation and expansion

Rossig et al., demonstrated that stimulation through the CAR alone was not sufficient to maintain proliferation and expansion of CAR-VST beyond four weeks (81). This proliferative deficit could however be overcome by stimulation with autologous EBV-LCL, highlighting the need for native TCR engagement for sustained CAR-VST activity. Savoldo et al., confirmed that VSTs stopped proliferating and progressively died when restimulation with LCL and IL-2 was halted, ruling out any potential for autonomous growth (82). Landmeier et al., reported that repeated stimulation with VZV lysates resulted in robust proliferation of CAR-VST whereas exposure to tumor target cells failed to induce similar proliferation (95). The requirement for continuous antigen and cytokine stimulations to maintain CAR-VST proliferation further emphasize the importance of the TCR signaling pathway.

##### 5.2.1.2 Impact of native TCR signaling on CAR-VST functionality

Beyond proliferation, different functional improvements were observed secondary to viral triggering. Specifically studies with CAR-VST after TCR engagement have demonstrated: (i) an increased expression of the CAR (85), both in CD4+ and CD8+ populations (84), (ii) an enhanced anti-tumor activity (92, 93), and (iii) a rescue of anti-tumoral dysfunction (89). Specifically, Tanaka et al., showed that VZV pepmix-loaded DCs could restore the antitumor activity of GD2.CAR-VZVSTs rendered dysfunctional by the tumor, suggesting that VZV vaccination could be leveraged to recover the function of CAR-VST cells through TCR stimulation.

In conclusion, the CAR and native TCR cooperate in enhancing the therapeutic potential of CAR-VST by ensuring robust and sustained immune responses. Specifically, the native TCR signaling is essential for the providing survival, proliferation, and expansion of CAR-VST.

#### 5.2.2 Use of costimulatory domains (CD28, 4-1BB)

The native TCR/CD3 complex, upon engagement with its cognate antigen, provides the primary activation signal (Signal 1). However, a second signal (Signal 2) mediated by costimulatory molecules such as CD28 or 4-1BB is required for full activation, and to avoid anergy or apoptosis. Cytokines production (Signal 3) is also crucial to maintain T cell proliferation and survival. Thus, the coordinated sequence of these signaling paths is pivotal for the function of T cells in general and of CAR T cells in particular.

The role played by signal 2 has been clearly demonstrated with VST. EBVST expressing the GD2-CAR (first generation) outperformed CAR T cells lacking costimulatory endodomains, highlighting the critical role of costimulation in enhancing T cells efficacy. With the incorporation of costimulatory endodomains into CARs for T cells becoming standard of practice, second generation CARs have also been used to transduce VST.

Altvater et al., formally compared in EBVST effector memory T cells first and second generation CARs, namely GD2.ζ and GD2.CD28ζ CAR (94) and observed similar dual cytotoxicity and comparable IFN-γ secretion. Interestingly no expansion of CAR-VST in response to antigen-expressing tumor cells was observed.

##### 5.2.2.1 CD28 versus 4-1BB

While the optimal costimulatory signal remains a topic of discussion, the majority of CAR-VST reported to date include a single CD28 co-stimulatory molecule. The report by Omer et al. is currently the only study that compares CD28 and 41BB signals in CAR-VSTs (86). The study evaluated in VZVST and EBVST first and second-generation GD2.CAR containing costimulatory endodomains derived from 4-1BB or CD28. The team found that a GD2.CAR containing both CD28 and CD3ζ chain (GD2.CD28ζ) significantly enhanced the function of CAR-VST compared to GD2.CAR containing 4-1BB and ζ (GD2.4-1BBζ) or ζ alone (GD2.ζ). Specifically, GD2.CD28ζ CAR-VST exhibited higher proliferation and cytokine secretion in response to TCR stimulation, and better expansion when stimulated through the CAR. In contrast, transduction of EBVST and VZVST with GD2.4-1BBζ or GD2.ζ halted their proliferation and function. The frequency of viral antigen-reactive T cells decreased in GD2.ζ and GD2.41BBζ VSTs, indicating T cell dysfunction rather than a loss of antigen-specific T cells. GD2.ζ and GD2.41BBζ VSTs exhibited also higher frequencies of apoptotic cells and increased Fas expression compared to NT controls and GD2.CD28ζ-transduced VST. Moreover, GD2.4-1BBζ VSTs displayed a marked downregulation of the TCR α/β-chains, associated with a decreased response to viral antigens. This downregulation was paralleled by an increased cell size and a higher CD25 expression, indicating activation. The study found a strong correlation between the expression of activation markers and TCR α/β downregulation. Similarly, CD28 co-stimulation appeared crucial for optimal expansion and function of VST transduced with a different CAR (CD19.CAR).

Regarding the choice of costimulation molecules for effective activation, it should be noted that authorized CAR-T cell therapies mainly use the 4-1BB costimulation domain rather than CD28. 4-1BB, featured in commercial CAR-T products like Kymriah^®^ and Breyanzi^®^, is known for promoting T cell persistence and a long-term memory phenotype, which is crucial for sustained antitumor activity. Conversely, CD28, used in Yescarta^®^ and Tecartus^®^, is associated with rapid, potent T cell activation and functional cytotoxicity that leads to immediate tumor reduction but may also result in quicker T cell exhaustion. Even if the choice seems to depend on balancing the need for immediate efficacy versus long-term durability, there is no consensus CD28 or 41BB being the best costimulatory molecule (107, 108). Preclinical studies suggest that CD28-based CARs induce greater cytokine release compared to 4-1BB-based CARs, both domains confer similar antitumor activity in mouse models. Clinically, CAR-T cells with either domain have shown high efficacy in treating relapsed hematological malignancies, with no significant differences in antitumor activity. However, large clinical trials have reported higher rates of neurological toxicities with CD28, likely due to other factors. Further investigations should focus on directly comparing these costimulatory domains while controlling for confounding variables.

#### 5.2.3 Characterization of the final product

##### 5.2.3.1 TCR repertoire

While CAR expression introduces a new antigen specificity to T cells, it does not alter their existing TCR repertoire. Thus, the TCR diversity originally present in the VST is maintained. The engineering process does not promote the expansion of a single clone; rather, it adds a new receptor to an already diverse set of T cells. Maintaining a polyclonal TCR repertoire in CAR-VST is essential for their effectiveness against diverse antigens.

Nakazawa et al., demonstrated that HER2.CAR-VSTs retained a polyclonal TCR repertoire, as shown by GeneScan analyses, which revealed typical polyclonal patterns for TCRβ and TCRγ regions (85). Similarly, Wang et al., showed that neither CMV-specific TCR isolation nor CD19.CAR engineering resulted in clonal expansion, thereby preserving their broad Vβ usage (92, 93). This diverse TCR repertoire ensures that CAR-VSTs can target a wide array of antigens, which is essential for maintaining an effective and versatile immune response.

##### 5.2.3.2 CD4 and CD8 cells

As previously mentioned, VST generated through culture or sorting methods, typically consist in a polyclonal population that includes both CD4+ and CD8+ T cell subsets, which are important for the sustained antitumor and antiviral efficacy of the CAR-VST products. CD8+ T cells serve as the cytotoxic arm, directly eliminating target cells while CD4+ T cells provide essential helper functions, boosting the activation, proliferation, and survival of CD8+ T cells. In this way, authors showed important variations of the CD4+/CD8+ ratio in the final CAR-VST products, probably depending on the viral infection status of the donor. Furthermore, it is worth noting that some studies have demonstrated that a high CD4/CD8 CAR ratio, in autologous CD19 CAR T cell products, is associated with poorer post-CAR T outcomes (109). Interestingly, VST products for CMV and EBV are generally CD8 dominant (110, 111), which aligns with the potential therapeutic benefits of a lower CD4/CD8 ratio, supporting better outcomes in this context.

##### 5.2.3.3 Inducing naive and memory cells

Multiple studies have shown that CAR-VSTs predominantly exhibit effector memory phenotype, which is linked to their capacity for rapid response upon antigen re-exposure (90, 94, 95). As mentioned previously, the methods used for the generation of VST (co-culture or isolation of IFNγ secreting cells) lead to the enrichment in mature T cells. Moreover, the expansion of CAR-VST after transduction, skew their maturation of T cell subsets. A study highlighted the differences in memory potential based on the timing of CAR transduction (84). Early-transduced VST (day 3) had a higher percentage of Tcm (CD62L+ CCR7+), suggesting greater memory potential and better therapeutic efficacy compared to late-transduced VST (day 19), which were more differentiated and potentially less effective in the long term. However, it was also suggested in another study that TCR stimulation promotes a more favorable phenotype for long-term function and persistence. Indeed, CD19.CAR-CMVST, when stimulated through their native TCR with pp65pepmix-loaded autologous PBMCs, exhibited higher expression of genes linked to persistence and memory, such as KLF2, TCF7, and Lef1, compared to CAR stimulation alone (93). Optimized expansion protocols must be developed to promote the growth of less mature subsets.

Two unexplored aspects of CAR-VST optimization deserve attention: modulating the effector-to-memory phenotype and adjusting cellular metabolism to support long-lived memory subsets. Currently, CAR-VST products predominantly exhibit a Tem phenotype, irrespective of the production approach. Investigating the shift of this phenotype toward more immature subsets (Tcm or even Tscm) could enhance therapeutic durability and efficacy. This approach has been little explored except by using IL7-IL15 cytokine-cocktail and only within the context of CAR-T cells (112–114). Additionally, favoring a metabolic profile that promotes oxidative phosphorylation could help maintaining a Tcm or Tscm profile, as it is under investigation for CAR-T cells, potentially supporting sustained persistence and antitumor functionality (115–117). While studies on these approaches are lacking within the CAR-VST framework, they offer promising directions for future research.

##### 5.2.3.4 Exhaustion markers

In addition to an optimal memory phenotype, the expression of exhaustion markers is being evaluated to generate less exhausted cells, for a better long-term survival. Exhaustion markers such as PD-1, LAG-3, and TIM-3 are typically upregulated in T cells that have been exposed to chronic antigen stimulation, leading to a decline in their functional capacity. However, Wang et al., found that CD19.CAR-CMVST cells did not display elevated levels of exhaustion markers following TCR stimulation (93). Similarly, Landmeier et al., observed that expanded VZVST maintain a robust memory phenotype (95), further supporting the potentials of CAR-VST for prolonged therapeutic applications.

#### 5.2.4 Suicide gene as a safety system

In efforts to manage the safety of allogeneic CAR-VST therapies and control unforeseen toxicities, several approaches to control and eliminate these cells have been tested. Two notable strategies include the use of the inducible caspase-9 (iCasp9) suicide gene and cetuximab-mediated antibody-dependent cellular cytotoxicity (ADCC).

The first strategy allows for the selective induction of apoptosis of transduced cells upon administration of a small molecule dimerizer, effectively eliminating the CAR-VST in the event of severe toxicity or off-target effects. Caruana et al., demonstrated the incorporation of the iCasp9 suicide gene in CAR-VST (83). The second strategy take advantage of expressing a truncated version of the epidermal growth factor receptor (EGFRt) for cells to be targeted and eliminated by cetuximab, a monoclonal antibody that induces ADCC. However, studies suggest that the truncated EGFR system may have limited efficiency as a safety switch in the context of neutropenia (118). Furthermore, alternative systems, such as those based on CD20 mimotopes, have also been explored as potential elimination markers, offering additional safety mechanisms (119, 120). Wang et al., explored the use of cetuximab-mediated ADCC as a safety mechanism for CAR-VST (93).

#### 5.2.5 Vaccination

An added feature of CAR expressed on VST is the possibility to leverage on the naïve TCR for prolonged persistence. Restimulating CAR-VST with the appropriate vaccine represents a promising approach to control persistence and functionality of CAR-VST. Several groups have studied this synergy. By using home-made (i.e. influenza virus) or existing vaccines (CMV or VZV vaccines) to stimulate the native TCR, several teams showed continuous activation and expansion of CAR-VST, maintaining their expansion and effector functions while preventing exhaustion. Indeed, Wang et al. reported significant increase in the frequency of human T cells and CAR+ CMVpp65-tetramer+ bispecific T cells in vaccinated mice compared to controls (92). For instance, human T cells in pp65-challenged mice reached 5.6% ± 2.6%, compared to only 0.3% ± 0.1% in controls. These bispecific T cells were also more abundant in the spleen, indicating a potential homing property. Landmeier reported that CAR-VZVST re-expanded after re-exposure to booster doses of a VZV vaccine (95). Moreover, vaccine could sustain antitumor effects in a relapsed tumor model, indicating that the vaccine could maintain the efficacy even after initial tumor progression (92) and could lead to a higher rate of complete tumor clearance with improved survival outcomes of mice compared to the one treated with CAR-VST alone (91). Similarly, in the CMV-vaccine murine model of Caruana, 47% of mice were tumor-free in the vaccinated group, compared to only 12% in the control group (83). However, one study also highlighted a potential risk of cytokine release syndrome (CRS), evidenced by significantly elevated levels of human-specific IFN-γ and IL-6 in the serum of mice (92). In this study, Caruana et al., explored another way to enhance expansion and proliferation of CAR-VST. They investigated the role of CD40L and OX40L, ligands of 2 molecules, CD40 and OX40, expressed on activated T cells and implicated in the immunological synapse to boost APCs. They transduced K562 cells with lentiviral vectors encoding either human CD40L or OX40L or pp65/eGFP or the combination CD40L/pp65 and OX40L/pp65. They generated GD2.CAR-CMVST with CD28 co-stimulation molecule. They observed cooperation between CD40L, OX40L and pp65 antigen presentation, significantly enhancing the activation and antitumor responses of the CAR-VSTs *in vivo* (n=8) in a murine model of xenogenic tumor, thanks to the induction of APC maturation upon antigen processing.

#### 5.2.6 Immunogenicity et alloreactivity

The limited alloreactive repertoire of VST is the base for CAR-VST to provide effective antitumor activity without inducing severe GvHD, even when derived from partially HLA-matched donors (6, 71, 72).

However, in an allogeneic context, CAR-VST remain targetable by the recipient cells, undermining the long-term persistence and thus efficacy of an infused product. A recent *in vitro* study has proposed an original strategy to prevent recipient T cell-mediated killing of CAR-VST (87). Because CD30, in addition to its expression by tumors cells in Hodgkin lymphoma, anaplastic large cell lymphoma and human T cell leukemia virus type 1 + T cell lymphoma, is an activation marker highly upregulated by alloreactive T cells its targeting through a CAR could promote an anti-tumoral effect while at the same time eliminate recipient alloreactive T cells. CD30.CAR-EBVST have been tested in a Mixed Lymphocyte Reaction (MLR) co-cultured with allogeneic PBMC or primed alloreactive T cells (p-ART) to simulate an alloreactive immune response. Non-transduced (NT) EBVST and CD30.CAR-EBVST were eliminated while CD30.CAR-EBVST persisted, expanded and prevented p-ART expansion.

## 6 Clinical translation of CAR-VST

The following section evaluates the feasibility, safety profile and efficacy of CAR-VST in clinical settings.

### 6.1 Feasibility

Clinical trials have demonstrated the feasibility of manufacturing CAR-VST products at clinical scale level. For instance, in the MULTIPRAT clinical trial (NCT00840853) HLA compatible CAR-VST were generated in a GMP compliant grade from an allo-SCT donor and infused into patients with relapsed B-cell malignancies post-allo-SCT (N=8) (68). This first clinical trial ensured safety and reproducibility of the generation of CAR-VST for clinical applications.

In the study by Quach et al., a bank of seven CD30.CAR EBVST lines was successfully generated (66). Further research by Sun et al., optimized the production process by incorporating early transduction techniques (84). This optimization process ensured that a higher proportion of T cells maintained central memory phenotypes, crucial for long-term persistence and efficacy. This Good Manufacturing Practice manufacturing process is currently applied for two clinical trials (NCT00840853/MULTIPRAT and NCT01460901/STALLONe). The HERT-GBM trial also showed successful manufacturing of 16 products for all the treated patients.

Overall, studies showed that manufacturing process successfully generated CAR-VST that met all release criteria, including viability, transduction efficiency and sterility. However, the scalability of the CAR-VST manufacturing process remain a significant challenge, as current clinical trials have only been conducted with small cohorts of patients. Expanding production to treat larger patient populations will require overcoming substantial logistical and technical hurdles. Advances in cell therapy manufacturing, such as automated culture systems and standardized protocols, may mitigate these challenges.

### 6.2 Safety of CAR-VST

The safety of CAR-VST has been a central focus in clinical research, with early-phase trials such as NCT00840853 showing a favorable safety profile for donor-derived CD19.CAR-VST, with no reported infusion-related toxicities or cases of GvHD. The CAR-VSTs persisted in patients for a median of 8 weeks in the blood and up to 9 weeks at disease sites, all without inducing significant adverse events (68). In the trial NCT04288726, which investigated CD30.CAR-EBVST, the safety of allogeneic CAR-VSTs was further confirmed in 14 patients. The study observed minimal severe adverse effects, with only a few instances of reversible grade 4 cytopenia and mild CRS, which resolved without intervention. Importantly, no cases of GvHD were reported, even in patients who received multiple infusions, including those with HLA mismatches products (66). The absence of GvHD maybe attributed to the fact that alloreactive recipient T cells would upregulate the CD30 molecule, which would be also targeted by the CAR. Consequently, no immediate rejection of CAR-VST by recipient T cells was observed even after multiple infusions.

Overall, CAR-VST therapies have demonstrated a consistently favorable safety profile with minimal severe toxicities. Most of the trials reported no infusion-related toxicities, with manageable adverse effects resolving without treatment. A significant advantage of CAR-VST is their reduced risk of GvHD, as these VST are less likely to cause off-target effects. This safety profile makes CAR-VST a potentially safer alternative to conventional CAR-T, especially in allogeneic settings.

### 6.3 Efficacy of CAR-VST

The efficacy of CAR-VST has been investigated as secondary endpoint of few clinical trials. In the NCT00840853 reported by Cruz et al., efficacy of donor-derived CD19.CAR-VST in the treatment of B-cell malignancies that have relapsed post-allo-SCT (68). This Phase 1 study involved eight patients treated with escalating-doses of allogeneic CAR-VST infused 3 months to 13 years post-HSCT. Objective antitumor effects were observed in 2 out of 6 patients with active disease, and 2 additional patients remained disease-free after receiving the therapy while in remission. One patient relapsed after 4 months and a second developed a Richter syndrome after 8 weeks. The CD19.CAR-VST demonstrated a modest persistence of 8 weeks in the blood and transgene was detectable until 12 weeks. In cases of viral reactivation, CAR-VST expanded, highlighting the role of natural infection/virus reactivation as potential mechanism to boost CAR-T cell numbers *in vivo*. No expansion of CAR-VST was observed with AdV positive viremia for one patient. In this study, viral reactivation was less frequently observed because of the cell infusion occurring, for some patients, long after allo-SCT. In the study of Lapteva et al., the role of TCR stimulation in enhancing the expansion and function of single-dose CD19.CAR-VST was specifically investigated, particularly in the absence of prior cytoreductive chemotherapy, in patients in remission of B-cell ALL with no evidence of minimal residual disease (97). In absence of viral reactivation (N=5), CAR-VST did not expand. In contrast, in patients who experienced viral reactivation (N=3), there was an outstanding expansion of CAR-VST up to 30,000-fold. Interestingly, only EBV reactivated. This led to effective depletion of CD19+ B cells and suggests that viral reactivation plays the role of a potent trigger for CAR-T cell expansion, avoiding the need for cytoreductive chemotherapy in some cases and even in absence of MRD. Five out of 8 patients remained in remission 42 to 60 months post-treatment, with EBVST still detectable. A similar observation was reported by Rossig et al. in the CD19TPALL trial (NCT01195480) (102). The aim of this multi-center phase I/II study was to determine if EBV-directed vaccination could improve the persistence and efficacy of CD19.CAR-EBVST in pediatric ALL with molecular relapse post first allo-SCT, or prophylactically post-second allo-SCT. Overall, at one-month post-infusion, 5 out of 11 treated patients achieved CR, with 1 *de novo* CR and 4 in CR for a 12-months follow up. One patient achieved PR, demonstrating some degree of antitumor activity. Three patients maintained a stable disease (SD) for 8 weeks to 29 months while 3 patients showed no response to the treatment, highlighting variability in therapeutic efficacy. However, at a median follow-up of 12 months, 10 out of 11 patients relapsed, with three patients remaining alive (two with disease and one in CR for three years). Median persistence of CD19.CAR-EBVST was improved significantly with vaccination directed with EBV antigens: 0 day (range: 0-28) without vaccination compared to 56 days (range: 0-221) with vaccination (P=0.06).

As mentioned before, other targets than CD19 were also investigated in early phase clinical trial. Quach reported a trial studying CD30.CAR-EBVSTs in patients with CD30+ lymphomas. Fourteen patients with r/r Hodgkin’s lymphoma were treated using escalating doses of CD30.CAR-EBVSTs. Thirteen patients among fourteen were evaluable for responses. The overall response rate was 69.2%, with 5/10 patients achieving CR and 4 patients achieving PR. The efficacy appeared dose-dependent, with higher response rates observed at higher dose levels. This suggests that the therapeutic potential of these CAR-VSTs may be optimized by adjusting the dosing regimen (98). The durability of responses varied, with some patients achieving long-term remission. For instance, patient 10, who had bulky disease, responded to three separate infusions from the same donor line, indicating that repeated administrations can maintain or enhance therapeutic efficacy. The study proposed several explanations for the rapid disappearance of circulating cells, including elimination by alloreactive T cells, short-life cells or residency at the tumor sites.

As a summary, the clinical trials conducted on CAR-VST therapies have demonstrated both the feasibility and safety of this approach in treating various malignancies. These studies highlighted that CAR-VSTs can be successfully manufactured in early-phases to meet clinical-grade standards. Safety was attested by few adverse events of low grade and absence of GvHD. However, the efficacy of CAR-VST therapies has shown variability across different trials and patient populations. While some patients have achieved complete remission and long-term survival, others have experienced disease progression or relapse, indicating that the current efficacy of CAR-VST therapies is not uniform. Factors such as the persistence of CAR-VSTs in the blood, their expansion in response to viral reactivation, and their residency at tumor sites are critical to achieve sustained antitumor activity. Long-term efficacy was associated in some trials with the potential of combining TCR and CAR stimulation to enhance the durability of CAR-T cell responses, and the importance of concomitant TCR stimulated by viral antigens. The reported studies suggest that enhancing the durability and expansion of CAR-VSTs, particularly through strategies like viral reactivation or vaccination, could improve therapeutic outcomes.

Moving forward, optimizing the manufacturing process to ensure a higher proportion of Tcm, exploring vaccination strategies that enhance CAR-VST persistence and define dose regimens are key areas that could improve the efficacy of CAR-VST therapies. Additionally, expanding these trials to larger cohorts will be essential to fully understand the therapeutic potential and to refine the approach for broader clinical application.

## 7 Conclusion and perspectives

In recent years, VSTs have emerged as a promising platform for CAR-T cell therapy, following a period of reduced interest in the field. This resurgence is largely driven by the evolution of understanding of VST biology and the development of more refined techniques for their genetic modification and expansion. The use of CAR-VSTs offers a unique advantage due to the inherent antiviral properties of VSTs, which may enhance the persistence and functionality of the engineered T cells in a therapeutic setting. Although we reported academic experiences of CAR-VSTs, pharmaceutical companies are also developing their own program with CAR-VSTs. Indeed, Atara Biotherapeutics, under the guidance of Pierre Fabre, has been at the forefront of developing EBVSTs for treating EBV-associated malignancies. Their product, Ebvallo^®^ (tabelecleucel), approved by EMA is the first allogeneic T-cell immunotherapy for EBV-positive post-transplant lymphoproliferative disease (EBV+ PTLD). This disease commonly affects transplanted patients who receive immunosuppressive drugs to prevent graft rejection or GvHD. Ebvallo^®^ is used as a monotherapy for this rare lymphoproliferative disease, involving stored EBVSTs generated from immunized healthy donors. The therapy has an orphan drug status in Europe. According to recent studies, tabelecleucel has shown a clinical benefit in patients with r/r EBV+ PTLD, a population with few treatment options, while maintaining a favorable safety profile (121–123). Atara Biotherapeutics is currently developing an allogeneic CAR-EBVST incorporating CD28 and an additional costimulatory molecule. Future clinical investigations will give some insight about the long-term efficacy and safety of this promising therapy.

Despite these advances, the application of CAR-VSTs in an allogeneic setting presents significant challenges, particularly the risk of rejection. While CAR-VSTs have shown promise in a directed allogeneic context—where donor cells are partially matched to minimize immune incompatibility—*off-the-shelf* allogeneic CAR-VSTs face substantial hurdles due to the risk of rejection. To mitigate these risks, strategies such as targeting CD30, which is expressed on both tumor cells and activated immune cells, including alloreactive T cells, have been explored. This dual-targeting approach could potentially reduce the risk of rejection while maintaining antitumor efficacy. Another avenue being investigated is the genetic deletion of HLA molecules to make universal CAR-VSTs that are less likely to be rejected by the host immune system. Several studies have highlighted the feasibility of this approach, demonstrating that CAR-T cells with deleted HLA molecules can evade alloreactive immune responses, though this strategy is still in the early stages of development (4, 124).

In conclusion, CAR-VSTs are gaining renewed interest as a promising *off-the-shelf* immunotherapy option, primarily due to their ability to avoid GvHD and their potential for long-term persistence through viral restimulation. While these features make these cells particularly attractive, the challenge of rejection in HLA-incompatible settings remains a significant hurdle. Future research will need to focus on overcoming this barrier, potentially through innovative strategies like HLA deletion, to fully harness the therapeutic potential of CAR-VSTs in allogeneic contexts.

## Glossary

AdV: Adenovirus
AdVST: Adenovirus specific T cell
AEMPS: Spanish Agency of Medicines and Medical Devices
ALL: Acute Lymphoblastic Leukemia
Allo-SCT: allogeneic hematopoietic stem cell transplantation
APC: Antigen Presenting Cell
ATMP: Advanced Therapy Medicinal Product
B2M: β-2-microglobulin
CAR: Chimeric Antigen Receptor
iCas9: inducible CRISPR associated protein 9
CD: Cluster of differentiation
CMV: Cytomegalovirus
CMVST: Cytomegalovirus Specific T cell
CIK: Cytokine-Induced killer
CRS: Cytokine release syndrome
CRISPR: clustered regularly interspaced short palindromic repeats
DC: Dendritic Cell
DNA: Deoxyribonucleic acid
DNT: Double Negative T cell
EBV: Eptein Barr Virus
EBVST: Eptein Barr Virus Specific T cell
EGFRt: truncated Epidermal growth factor receptor
EMA: European Medicine Agency
EU: European Union
FDA: Food and Drug Administration
GD2: disialoganglioside
GR: Graft Rejection
GvHD: Graft versus Host Disease
HGBL: high-grade B-cell lymphoma
HLA: Human Leukocyte Antigen
IFN-γ: Interferon gamma
iNKT: invariant Natural Killer T cell
iPSC: Induced pluripotent stem cell
KIR: Killer cell immunoglobulin-like receptor
KO: Knock-out
LAG-3: Lymphocyte-activation gene 3
LCL: Lymphoblastoid Cell Line
MAIT: Mucosal-Associated Invariant T cell
MHC: Major Histocompatibility Complex
MP-1: influenza A Matrix Protein 1
MRD: Minimal residual disease
NK: Natural Killer cell
ORR: Objective Response Rate
p-ART: primed Alloreactive T cells
PBMC: Peripheral Blood Mononuclear Cell
PD-1: Programmed cell death 1
PTLD: Post-transplant lymphoproliferative disorder
rhIL: recombinant human Interleukin
r/r: Refractory or relapse
scFv: Single Chain Fragment Variable
shRNA: Small hairpin RNA
SOT: Solid Organ Transplantation
TALEN: Transcription Activator-Like Effector Nuclease
Tcm: Central memory T subset
TCR: T Cell Receptor
Tem: Effector memory T subset
TIM-3: T cell immunoglobulin and mucin domain-containing protein 3
TNF-α: Tumor Necrosis factor-Alpha
TRAC: T cell Receptor Alpha Constant
Tscm: Stem cell memory T subset
UCB: Umbilical cord blood
VST: Virus Specific T cell
VZV: Varicella Zoster Virus
VZVST: Varicella Zoster Virus Specific T cell
ZAP70: Zeta Chain of T Cell Receptor Associated Protein Kinase 70
ZFN: Zinc Finger Nucleases
